# Vagal nerve stimulation started just prior to reperfusion limits infarct size and no-reflow

**DOI:** 10.1007/s00395-015-0508-3

**Published:** 2015-08-26

**Authors:** André Uitterdijk, Tuncay Yetgin, Maaike te Lintel Hekkert, Stefan Sneep, Ilona Krabbendam-Peters, Heleen M. M. van Beusekom, Trent M. Fischer, Richard N. Cornelussen, Olivier C. Manintveld, Daphne Merkus, Dirk J. Duncker

**Affiliations:** Division of Experimental Cardiology, Department of Cardiology, Thoraxcenter, Erasmus MC, University Medical Center Rotterdam, PO Box 2040, 3000 CA Rotterdam, The Netherlands; Medtronic Research and Technology, Minneapolis, MN USA; Medtronic Bakken Research Center, Maastricht, The Netherlands

**Keywords:** Cardioprotection, Macrophages, Myocardial infarction, Neutrophils, Reperfusion injury, Swine

## Abstract

Vagal nerve stimulation (VNS) started prior to, or during, ischemia has been shown to reduce infarct size. Here, we investigated the effect of VNS when started just prior to, and continued during early, reperfusion on infarct size and no-reflow and studied the underlying mechanisms. For this purpose, swine (13 VNS, 10 sham) underwent 45 min mid-LAD occlusion followed by 120 min of reperfusion. VNS was started 5 min prior to reperfusion and continued until 15 min of reperfusion. Area at risk, area of no-reflow (% of infarct area) and infarct size (% of area at risk), circulating cytokines, and regional myocardial leukocyte influx were assessed after 120 min of reperfusion. VNS significantly reduced infarct size from 67 ± 2 % in sham to 54 ± 5 % and area of no-reflow from 54 ± 6 % in sham to 32 ± 6 %. These effects were accompanied by reductions in neutrophil (~40 %) and macrophage (~60 %) infiltration in the infarct area (all *p* < 0.05), whereas systemic circulating plasma levels of TNFα and IL6 were not affected. The degree of cardioprotection could not be explained by the VNS-induced bradycardia or the VNS-induced decrease in the double product of heart rate and left ventricular systolic pressure. In the presence of NO-synthase inhibitor LNNA, VNS no longer attenuated infarct size and area of no-reflow, which was paralleled by similarly unaffected regional leukocyte infiltration. In conclusion, VNS is a promising novel adjunctive therapy that limits reperfusion injury in a large animal model of acute myocardial infarction.

**Electronic supplementary material:**

The online version of this article (doi:10.1007/s00395-015-0508-3) contains supplementary material, which is available to authorized users.

## Introduction

The single most effective therapy to limit myocardial infarct size and improve clinical outcome after acute myocardial infarction is early coronary reperfusion via primary percutaneous coronary intervention [41, 43]. However, despite its overall benefits, reperfusion therapy has been shown to result in additional cardiomyocyte death, termed lethal reperfusion injury [12, 19, 52], and in microvascular obstruction, termed no-reflow [38]. No-reflow refers to inadequate myocardial reperfusion following opening of the culprit coronary lesion, despite lack of angiographic evidence of epicardial vessel obstruction and may be present in 30–40 % of patients [34]. Since both infarct size [30] and extent of no-reflow [17, 33, 38] are independent predictors of clinical outcome, strategies to limit these two components of reperfusion injury have significant therapeutic potential.

A novel strategy against ischemia–reperfusion injury was proposed by Katare et al. [21], who demonstrated that vagal nerve stimulation (VNS) limited infarct size in rats. This initial observation was confirmed in subsequent studies in rats [8, 25, 48, 54], mice [22] and swine [40]. In these studies, VNS was started either before [8, 21, 54], at the very onset of [22, 25, 40], or halfway through [39, 48] the period of ischemia. In contrast, a recent study reported that an intermittent VNS protocol failed to attenuate infarct size when started at the very onset of reperfusion [39]. In view of the critical importance of the presence of an intervention during the golden first minute(s) of reperfusion [42], we hypothesized that continuous VNS started just prior to reperfusion might be effective against reperfusion injury. Consequently, the first aim of the present study was to investigate the effects of continuous VNS starting 5 min prior to reperfusion [to ensure full VNS during the first minute(s) of reperfusion] on infarct size in a large animal model of ST-elevation myocardial infarction (STEMI). Moreover, since none of the aforementioned studies assessed the effects of VNS on no-reflow, despite its prognostic potential in the clinical setting [17, 33, 38], the second aim of the present study was to investigate the effects of VNS on no-reflow.

The mechanisms underlying the cardioprotective effects of VNS against myocardial necrosis are incompletely understood, but have been proposed to include activation of muscarinic receptors [40] and blunting of the inflammatory response [48]. Consequently, the second aim of the present study was to investigate the potential mechanisms underlying the effects of VNS on infarct size and no-reflow, in particular anti-inflammatory actions, including circulating inflammatory cytokines and myocardial neutrophil and macrophage infiltration, and the nitric oxide synthase (NOS) signaling pathway.

## Materials and methods

Studies were performed in 63 Yorkshire × Landrace swine (~55 kg) of either sex, in accordance with the “Guiding Principles in the Care and Use of Laboratory Animals” as approved by the Council of the American Physiological Society, and with approval of the Animal Care Committee of the Erasmus MC Rotterdam.

### Animal preparation

Swine were sedated with ketamine (20 mg/kg, i.m.) and midazolam (1 mg/kg, i.m.), anesthetized with pentobarbital (15 mg/kg i.v.), intubated and mechanically ventilated with O_2_ and N_2_ (1:3 v/v), and instrumented as previously described [26, 44]. Catheters were placed in the external jugular vein for maintenance of anesthesia (pentobarbital, 10–15 mg/kg/h) and infusion of physiological saline, and the left femoral artery for measurement of arterial blood pressure. A Swan–Ganz catheter was inserted into the left femoral vein and advanced into the pulmonary artery for body core temperature monitoring. A micromanometer-tipped catheter (SPC-3705, Millar Instruments, Houston, USA) was inserted into the right carotid artery and advanced into the left ventricle (LV) for measurement of LV pressure and its first derivative (LVdP/dt). Following sternotomy, an electromagnetic flow probe (P18, Skalar Medical, Delft, The Netherlands) was placed around the ascending aorta to measure cardiac output, and a transit-time flow probe (3SB, Transonic Systems, Ithaca, USA) was placed around the left anterior descending coronary artery (LAD) for measurement of coronary blood flow. A suture was placed around the LAD, just distal to its first diagonal branch, to enable coronary artery occlusion (CAO) during the experimental protocol. Regional myocardial function was measured in the area at risk and remote myocardium using two pairs of ultrasonic crystals (P/N SL5-2, Triton Technology Inc., San Diego, USA) placed in the mid-myocardium [11, 44]. A fluid-filled catheter was placed in the left atrium through a purse-string suture into the auricle for measurement of left atrial pressure. For coronary venous blood sampling, a custom fluid-filled catheter was placed in the anterior coronary vein draining the perfusion territory of the LAD. Heparin (5000 i.u./h, i.v.) was administered throughout the experiment to prevent coagulation.

Custom-made, self-coiling cuff electrodes were placed around both the left and right vagal nerves for VNS. After completion of instrumentation, the cardiac vagal nerve reserve (maximal percent reduction in heart rate) was tested in both vagal nerves (left and right) in 1 min intervals by increasing voltages (Medtronic 3625 test simulator; 2.0–10.5 V; 10 mA; pulse width 0.3 ms; 25 Hz). The maximum effect of VNS was reached during stimulation with 10.5 V. Maximum heart rate reductions during therapy, produced by stimulation of either left (15 ± 2 %) or right vagal nerve (18 ± 3 %), were not different (*p* = 0.41). Maximum heart rate reductions at pre-ischemia baseline were also comparable between VNS (23 ± 2 %) and sham (21 ± 3 %) groups (*p* = 0.69).

### Experimental protocols

#### Cardioprotection by VNS

After 30 min of stabilization, swine were subjected to the experimental protocol. Systemic and coronary hemodynamics and regional myocardial function were continuously recorded. Blood samples were obtained from the aorta and anterior interventricular coronary vein at several time points for measurement of PO_2_ (mmHg), PCO_2_ (mmHg), pH, O_2_ saturation (%), hemoglobin (Hb, in grams per 100 ml) and lactate (μmol/l) (Acid–Base Laboratory model 800, Radiometer, Copenhagen, Denmark). Body core temperature was monitored and maintained between 37.0 and 38.0 °C throughout the experimental protocol [13]. Swine were subjected to a 45-min CAO followed by 120 min of reperfusion. Swine encountering ventricular fibrillation (VF) were allowed to complete the protocol if conversion to sinus rhythm was successful within 2 min after onset of VF. Swine were randomly assigned to either sham treatment (*n* = 13) or VNS alone (*n* = 17). VNS was started 5 min before the end of CAO and continued until 15 min into reperfusion. All VNS animals were initially subjected to stimulation of the left vagal nerve. In five animals, in which the reduction in heart rate during the first minute was less than 10 %, right vagal nerve stimulation was applied during the remainder of the 20-min stimulation protocol.

#### Role of NO-synthase

To investigate the role of NO signaling in the protection by VNS, we performed a second series of experiments in which we studied three additional groups of swine. Animals underwent the identical 45-min CAO and 120-min reperfusion protocol, as outlined above, while receiving (1) control–sham treatment (*n* = 11), (2) NO-synthase inhibition, using *N*^ω^-nitro-l-arginine (LNNA, Sigma, Zwijndrecht, The Netherlands) 10 mg/kg i.v. 30 min before CAO [27], and sham treatment (*n* = 10), or (3) LNNA and VNS (*n* = 12).

### Infarct size and area of no-reflow

At the end of 120 min of reperfusion, a fluid-filled catheter was inserted into the coronary artery, distal to the site of occlusion, to administer 5 ml of a 4 % (w/v) thioflavin-S (Sigma) solution to determine the area of no-reflow [36, 45]. The coronary artery was re-occluded and the area at risk was delineated by intra-atrial infusion of 40 ml of 15 % (w/v) Evans Blue [13, 26]. Then, the heart was excised, the LV was isolated and cut into five transverse slices of equal thickness, and slices were weighed. After the area at risk and area of no-reflow (using UV light) of each slice were demarcated on an acetate sheet, the slices were incubated in 3 % (w/v) 2,3,5-triphenyltetrazolium chloride at 37 °C for 15 min to stain metabolically active tissue [13] and delineate infarcted area from non-infarcted area. The red stained non-infarcted area was also traced onto the sheet. The area at risk, area of no-reflow, and infarct area from each of the five transverse slices were determined and summed. Myocardial infarct size (IS) was defined as the ratio of the summed infarct areas (IA) and summed areas at risk (AR): IS = IA/AR × 100 % [13, 26]. Area of no-reflow (NR) was defined as the ratio of the summed no-reflow areas (NA) and summed infarct areas: NR = NA/IA × 100 % [36].

### Systemic inflammation

Plasma isolated from arterial blood taken at baseline, prior to CAO, and the first hour of reperfusion was analyzed by ELISA according to the manufacturer’s instructions for porcine inflammatory markers TNFα and IL6 (R&D Systems, Abingdon, UK).

### Regional inflammation

Infarct area with either reflow or no-reflow and remote non-area at risk (posterior wall) LV tissue was fixed in 4 % buffered formaldehyde and embedded in paraffin. To identify the acute influx of immune cells, sections of 4 µm were stained for neutrophils (Azurocidin, mouse anti human, 1:100, Abnova, Heidelberg, Germany) following antigen retrieval (10 min citrate buffer boil (pH 6)) and for macrophages (MAC387, mouse anti macrophage, 1:100, Abcam, Cambridge, United Kingdom). Staining was visualized using rabbit anti-mouse secondary antibodies (1:100, horseradish peroxidase label, DAKO, Heverlee, Belgium) with 3,3′-diaminobenzidine (DAB, DAKO) and H_2_O_2_ as chromogen. Primary antibodies were omitted as a negative control. Three randomly selected high power fields (90,000 µm^2^ per field) per section were morphometrically quantified in a blinded manner using dedicated software (Clemex Vision PE, version 6.0.010A, Clemex Technologies inc, Longueuil, Canada). Data were expressed as the number of cells/mm^2^.

### Data and statistical analysis

Hemodynamic and LV global and regional function data were recorded and analyzed, and myocardial oxygen and lactate consumption, and systolic and post-systolic shortening were calculated, as previously described [26, 44].

Inter-group differences in AR/LV, IA/AR and NR/IA were analyzed using unpaired *t* test or one-way ANOVA followed by Student–Newman–Keuls (SNK) post hoc testing, as appropriate. Hemodynamics and LV function were analyzed using two-way (time × treatment) ANOVA followed by SNK test. Global and regional inflammation were also analyzed using two-way (treatment × location) ANOVA followed by SNK test. Values are expressed as mean ± SEM. *p* < 0.05 (two tailed) was considered to be statistically significant.

## Results

### Mortality

In the first series of experiments, 26 out of 30 swine (11 out of 13 sham, 15 out of 17 VNS) encountered VF during the 45 min of CAO (all prior to VNS or sham). Seven out of these 26 swine (3 out of 11 sham, 4 out of 15 VNS) could not be successfully converted to sinus rhythm within 2 min and were therefore excluded from further study. The numbers of animals that encountered VF but could be converted to sinus rhythm within 2 min and completed the experimental protocol were not different (*p* = 0.76) between sham (8 out of 10 swine; 80 %) and VNS (11 out of 13 VNS; 85 %). Moreover, the average number of VF episodes before the start of VNS (1.8 ± 0.4) or sham (1.4 ± 0.4) treatment in these animals was also similar between groups (*p* = 0.45). Importantly, VF did not occur during VNS or the corresponding sham period or after 15 min of reperfusion, except for one sham animal that encountered VF at 11 and 15 min of reperfusion.

In the second series of experiments, 11 out of 11 (100 %) control–sham, and 17 out of 22 (77 %) LNNA-treated swine (*p* = 0.09) encountered VF during the 45 min of CAO (all prior to sham or VNS treatment). However, of the animals that encountered VF, 53 % (9 out of 17) LNNA-treated swine versus 27 % (3 out of 11) control–sham animals (*p* = 0.19) could not be successfully converted to sinus rhythm within 2 min and hence did not complete the experimental protocol. Finally, two swine (LNNA + VNS) developed progressive pump failure during reperfusion and could not complete the experimental protocol.

### Hemodynamics and global and regional LV function

Coronary artery occlusion of the LAD produced complete loss of regional systolic segment shortening, accompanied by an increase in post-systolic shortening in the LAD perfusion territory, which resulted in decreases in LV systolic pressure, LVdP/dt_*P*40_, stroke volume, cardiac output, and mean aortic pressure (Table 1). Global and regional LV function remained severely depressed during the 120-min reperfusion period.

**Table 1:** Systemic hemodynamics and global and regional left ventricular function

	Baseline	Coronary artery occlusion		Reperfusion	
		40 min	45 min	15 min	120 min
*Systemic hemodynamics*					
HR (bpm)					
Sham	111 ± 6	117 ± 8	116 ± 8	113 ± 7	109 ± 5
VNS	103 ± 4	107 ± 4	87 ± 4*^†‡^	89 ± 3*^†‡^	109 ± 4
MAP (mmHg)					
Sham	94 ± 3	82 ± 3*	80 ± 3*	76 ± 4*	73 ± 3*
VNS	91 ± 3	77 ± 5*	65 ± 5*^†^	65 ± 4*^†^	71 ± 3*
CO (l/min)					
Sham	4.2 ± 0.3	3.4 ± 0.1*	3.3 ± 0.1*	3.3 ± 0.2*	2.8 ± 0.1*^†^
VNS	3.5 ± 0.1	2.9 ± 0.2*	2.3 ± 0.1*^†‡^	2.4 ± 0.2*^†^	2.5 ± 0.1*^†^
SV (ml)					
Sham	38 ± 2	30 ± 2*	30 ± 2*	30 ± 2*	26 ± 2*
VNS	34 ± 2	27 ± 1*	27 ± 2*	28 ± 2*	23 ± 2*
*Global and regional LV function*					
LVSP (mmHg)					
Sham	109 ± 3	95 ± 3*	93 ± 3*	90 ± 3*	85 ± 4*
VNS	106 ± 2	90 ± 5*	78 ± 5*^†^	79 ± 4*^†^	85 ± 3*
LVdP/dt_*P* = 40_ (mmHg/s)					
Sham	1700 ± 160	1340 ± 80*	1320 ± 60*	1330 ± 80*	1120 ± 60*
VNS	1600 ± 70	1250 ± 80*	1060 ± 70*	1100 ± 80*	1110 ± 50*
LVEDP (mmHg)					
Sham	14 ± 1	16 ± 1	15 ± 1	16 ± 1	15 ± 1
VNS	13 ± 1	17 ± 1*	16 ± 1*	18 ± 1*	16 ± 1*
SS_LAD_ (%)					
Sham	15.1 ± 1.4	−7.2 ± 1.0*	−5.9 ± 0.7*	0.7 ± 0.4*	0.2 ± 0.6*
VNS	21.2 ± 1.2	−8.8 ± 1.3*	−7.0 ± 1.2*	2.0 ± 1.2*	0.1 ± 1.3*
SS_LCx_ (%)					
Sham	17.2 ± 2.2	12.8 ± 2.0*	12.8 ± 1.9*	13.9 ± 1.3*	10.8 ± 1.1*
VNS	17.2 ± 1.4	17.4 ± 1.2	17.7 ± 1.3	18.1 ± 1.4	15.4 ± 1.2
PSS_LAD_ (%)					
Sham	0.9 ± 0.2	10.7 ± 1.3*	9.0 ± 1.1*	2.3 ± 0.4^†^	2.9 ± 0.7^†^
VNS	1.2 ± 0.4	15.0 ± 1.1*	12.5 ± 1.1*^†^	4.3 ± 1.3*^†^	5.0 ± 1.4*^†^
PSS_LCx_ (%)					
Sham	0.5 ± 0.2	1.2 ± 0.5	1.1 ± 0.5	0.3 ± 0.1	0.7 ± 0.2
VNS	0.8 ± 0.2	1.1 ± 0.3	1.2 ± 0.1	1.0 ± 0.3	1.3 ± 0.6

Data are mean ± SEM; sham group, *n* = 10; VNS group, *n* = 13. Except for CO (*p* = 0.004) and SS_LAD_ (*p* < 0.001), no differences between groups at baseline

*CO* cardiac output, dP/dt_*P* = 40_ rate of rise in left ventricular (LV) pressure during LV pressure of 40 mmHg, *HR* heart rate, *LVEDP* left ventricular end-diastolic pressure, *LVSP* left ventricular systolic pressure, *MAP* mean arterial pressure, *PSS* post-systolic shortening, *SS* systolic shortening, *SV* stroke volume, *VNS* vagal nerve stimulation

* *p* < 0.05 versus corresponding baseline;^ †^ *p* < 0.05 versus corresponding 40 min CAO;^ ‡^ *p* < 0.05 change by VNS versus corresponding sham

The 18 ± 2 % decrease in heart rate produced by VNS was associated with a 19 ± 2 % decrease in cardiac output and a 16 ± 4 and 13 ± 3 % decrease, respectively, in mean aortic and LV peak systolic pressure at 45 min of CAO (Table 1). VNS did not alter LVdP/dt_*P*40_, LV end-diastolic pressure, or regional systolic and post-systolic segment shortening, indicating that VNS did not affect global or regional LV function. These effects of VNS were maintained during reperfusion, although heart rate showed partial escape from VNS during the first 5 min of reperfusion, which was likely due to the occurrence of premature ventricular contractions. After the termination of VNS, systemic hemodynamics and global and regional LV function were no longer different between VNS and sham swine.

### Coronary blood flow and myocardial metabolism

During reperfusion, transient increases in coronary blood flow and coronary vascular conductance were observed, together with a transient reversal from lactate consumption to production and a sustained depression of oxygen consumption (Table 2).

**Table 2:** Regional coronary blood flow and myocardial metabolism

	Baseline	Reperfusion	
		15 min	120 min
CBF (ml/min)			
Sham	20 ± 4	34 ± 6*	22 ± 3
VNS	15 ± 1	26 ± 4*	21 ± 2
CBF (ml/beat)			
Sham	0.17 ± 0.03	0.30 ± 0.05*	0.21 ± 0.03
VNS	0.14 ± 0.01	0.30 ± 0.06*	0.20 ± 0.02
MVO_2_ (µmol/min)			
Sham	90 ± 20	36 ± 6*	28 ± 4*
VNS	67 ± 7	29 ± 5*	23 ± 3*
MVO_2_ per beat (µmol/beat)			
Sham	0.77 ± 0.13	0.32 ± 0.05*	0.26 ± 0.03*
VNS	0.65 ± 0.13	0.33 ± 0.05*	0.21 ± 0.03*
CVC (ml/min/mmHg)			
Sham	0.21 ± 0.05	0.43 ± 0.05*	0.31 ± 0.04
VNS	0.16 ± 0.02	0.32 ± 0.05*	0.29 ± 0.02*
Lactate production (µmol/l/min)			
Sham	−14 ± 5	10 ± 5*	−7 ± 2
VNS	−7 ± 1	10 ± 2*	−4 ± 2
O_2_ Extraction (%)			
Sham	73 ± 2	19 ± 2*	21 ± 3*
VNS	71 ± 2	19 ± 2*	17 ± 2*
Lactate extraction (%)			
Sham	24 ± 4	−13 ± 6*	9 ± 3
VNS	24 ± 5	−15 ± 3*	6 ± 3*

Data are mean ± SEM; sham group, *n* = 10; VNS group, *n* = 13

*CBF* coronary blood flow, *CVC* coronary vascular conductance (CBF/MAP), *MVO*_*2*_ myocardial oxygen consumption, *O*_*2*_ *extraction* myocardial oxygen extraction

* *p* < 0.05 versus corresponding baseline;^ †^ *p* < 0.05 change by VNS versus corresponding sham

There were no significant differences between VNS and sham swine in the responses of coronary blood flow, coronary vascular conductance, myocardial consumption, and extraction of oxygen or lactate during early reperfusion (Table 2; Supplemental Figure S1).

### Infarct size and area of no-reflow

**Fig. 1:**
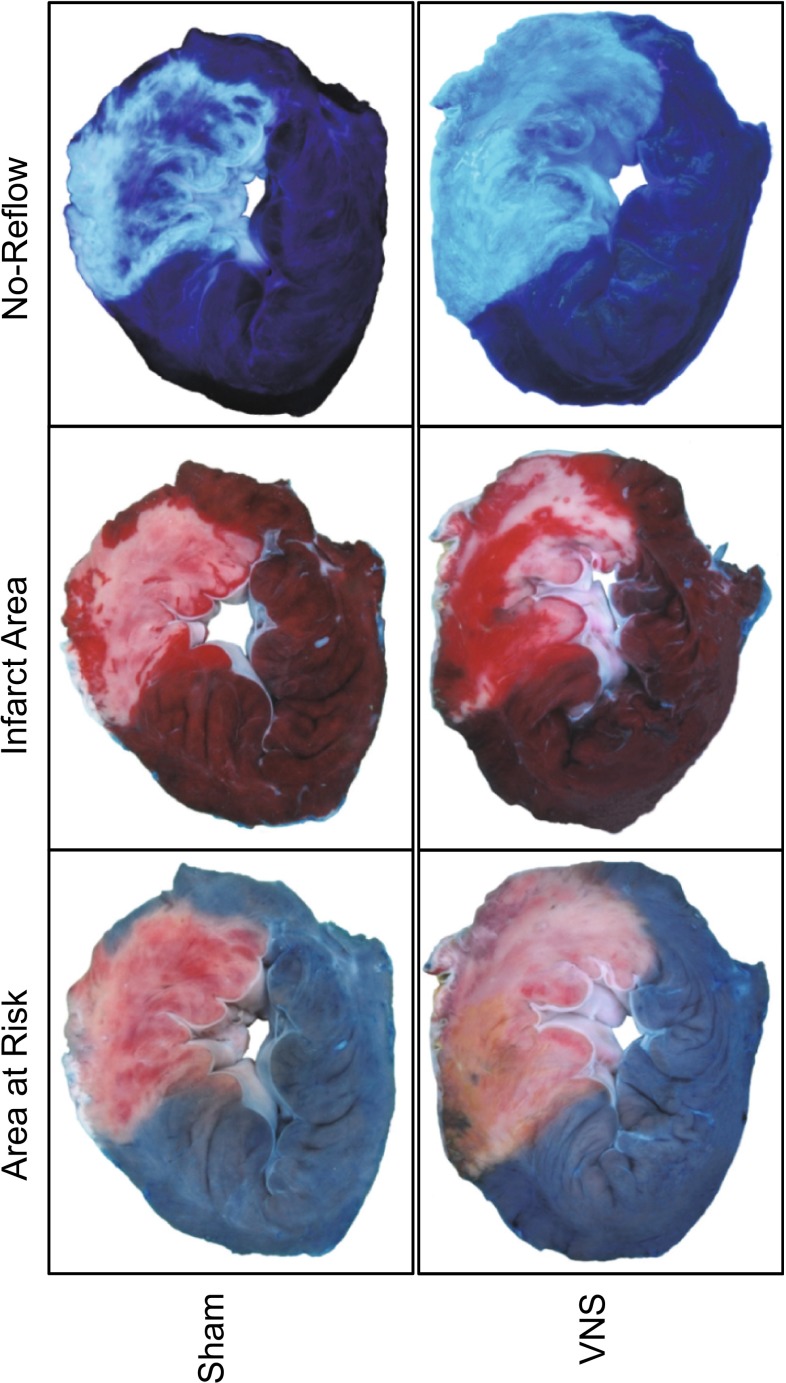
Typical examples of effects of VNS on infarct size and no-reflow. No-reflow is typically located subendocardially

**Fig. 2:**
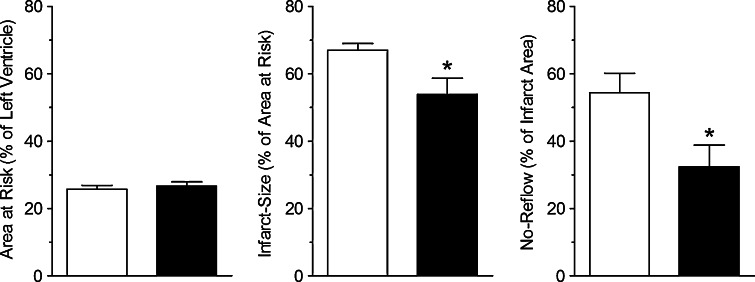
Effects of VNS on infarct size and no-reflow of sham (*white square*) and VNS (*black square*) animals. Data are mean ± SEM; *p < 0.05 versus sham

Ligation of the LAD distal to the first diagonal branch resulted in an average area at risk of 26 ± 1 % of the LV (Figs. 1, 2) and did not differ between sham (26 ± 1 %) and VNS (27 ± 1 %; *p* = 0.60). CAO of 45 min resulted in an infarct size in sham swine of 67 ± 2 % of the area at risk and a no-reflow area of 54 ± 6 % of the infarct area. VNS reduced infarct size to 54 ± 5 %, which was accompanied by a decrease in no-reflow area to 32 ± 6 % (both *p* = 0.03). There were no differences between swine undergoing left or right VNS, in terms of infarct size (left 52 ± 7 vs. right 58 ± 7 %, *p* = 0.56) or no-reflow area (left 32 ± 9 vs. right 32 ± 10 %, *p* = 1.00).

### Systemic and regional inflammation

**Fig. 3:**
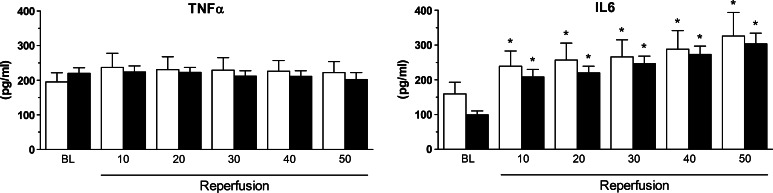
Effects of VNS on early systemic markers for inflammation. Shown are the effects of treatment on TNFα and IL6 levels in circulating plasma of sham (*white square*) and VNS (*black square*) animals. Data are mean ± SEM; *p < 0.05 versus corresponding baseline

Systemic TNFα levels did not rise in the early phase after reperfusion, while IL6 increased significantly compared to baseline but no differences between treatment groups were found (Fig. 3).

**Fig. 4:**
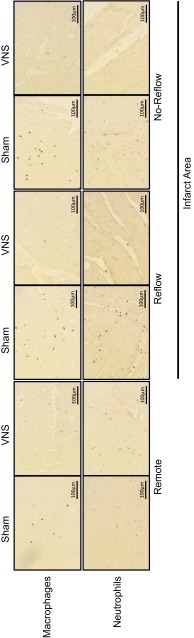
Typical examples of effects of VNS on regional leukocyte presence. Shown are macrophage and neutrophil influx in remote, infarct–reflow and infarct–no-reflow tissue of sham and VNS animals

**Fig. 5:**
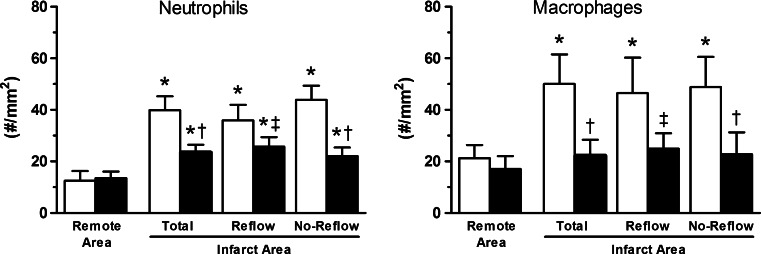
Effects of VNS on regional inflammation. Shown are the effects of treatment on neutrophil and macrophage numbers in remote, total infarct, infarct–reflow and infarct–no-reflow tissue of sham (*white square*) and VNS (*black square*) animals. Data are mean ± SEM; *p < 0.05 versus corresponding remote area; ^†^ *p* < 0.05 versus corresponding sham; ^‡^ *p* < 0.10 versus sham

In control animals, neutrophil and macrophage numbers were increased in the infarct area as compared to the remote myocardium, measured after 120 min of reperfusion. VNS attenuated neutrophil as well as macrophage influx into the infarct area (Figs. 4, 5).

### Role of NO-synthase

Infusion of LNNA in 11 swine resulted in an 18 ± 5 % increase in mean arterial pressure from 91 ± 4 to 108 ± 8 mmHg, which was accompanied by a 9 ± 1 % decrease in heart rate from 107 ± 7 to 98 ± 6 bpm, and an 18 ± 2 % decrease in cardiac output from 3.9 ± 0.1 to 3.2 ± 0.1 l/min (all *p* < 0.01). LNNA did not affect the responses to ischemia and reperfusion of systemic hemodynamics and global and regional LV function (Supplemental Table S1), or coronary hemodynamics and myocardial metabolism (Supplemental Table S2). Importantly, LNNA did not modify the hemodynamic, functional and metabolic responses to VNS (Supplemental Tables S1 and S2).

LNNA had no effect on infarct size, no-reflow area, or leukocyte influx (Table 3). However, LNNA prevented the VNS-induced limitation of infarct size (71 ± 6 % in LNNA + VNS vs. 65 ± 5 % in LNNA + sham) and no-reflow (22 ± 5 % in LNNA + VNS vs. 13 ± 4 % in LNNA + sham), which was paralleled by the prevention of VNS-induced reduction in leukocyte influx (Table 3).

**Table 3:** Effects of NO-synthase inhibition

	Control + sham	LNNA + sham	LNNA + VNS
	*n* = 8	*n* = 5	*n* = 6
Area at risk (% LV)	26 ± 2	25 ± 3	27 ± 2
Infarct size (% AR)	50 ± 4	65 ± 5	71 ± 6
No-reflow (% AR)	4 ± 1	8 ± 2	16 ± 4
No-reflow (% IA)	9 ± 2	13 ± 4	22 ± 5
*Cell influx in infarct area*			
Neutrophils (#/mm^2^)	12 ± 2	7 ± 2	25 ± 6^†^
Macrophages (#/mm^2^)	58 ± 13	86 ± 35	62 ± 17

Data are mean ± SEM

*AR* area at risk, *IA* infarct area, *LV* left ventricle, *n* number of animals that survived and completed the experimental protocol

* *p* < 0.05, LNNA + sham versus control + sham;^ †^ *p* < 0.05, LNNA + sham versus LNNA + VNS

### Role of changes in hemodynamic determinants of myocardial oxygen demand

**Fig. 6:**
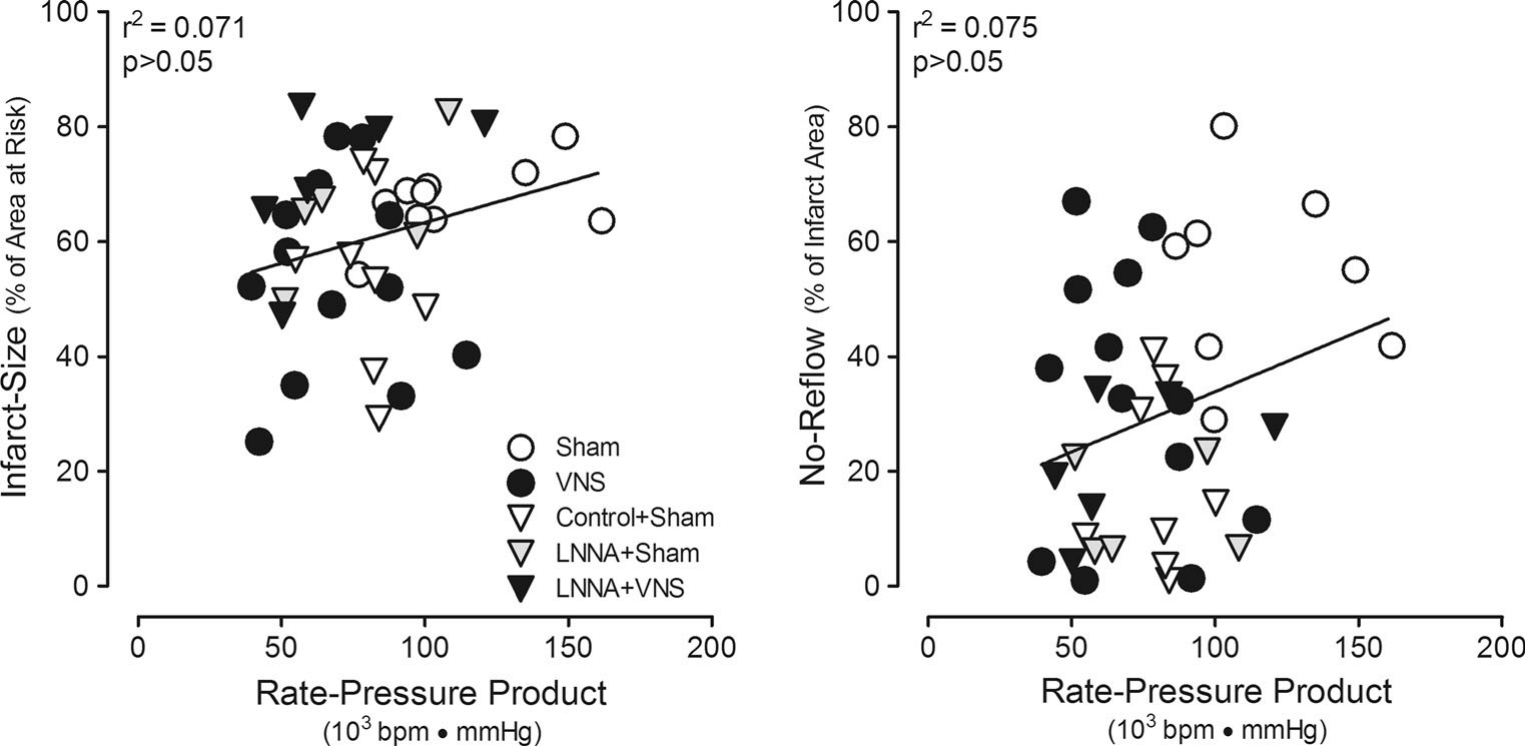
Lack of correlation between the double product of heart rate and systolic arterial pressure and infarct size (*left panel*) and no-reflow (*right panel*) at onset of reperfusion (45 min CAO)

Since VNS produced decreases in heart rate and LV systolic pressure, it could be argued that changes in the hemodynamic determinants of myocardial oxygen demand contributed to the cardioprotection by VNS. However, the observation that LNNA abolished the VNS-induced cardioprotection at a time when the hemodynamic effects of VNS were unmitigated suggests that the cardioprotective effects of VNS were independent of its hemodynamic effects. Indeed, regression analysis did not reveal a significant correlation between either infarct size (*r*^2^ = 0.071) or no-reflow area (*r*^2^ = 0.075), and the double product of heart rate and systolic arterial pressure (Fig. 6).

## Discussion

The present study is the first to investigate the effect of VNS started just prior to and continued during early reperfusion on both infarct size and extent of no-reflow in a large animal model of STEMI, using a clinically translational protocol. The main findings were that (1) VNS significantly limited infarct size and extent of no-reflow; (2) these effects were accompanied by reductions in regional infiltration of neutrophils and macrophages; (3) Inhibition of NO-synthase prevented the cardioprotection by VNS against necrosis, no-reflow and leukocyte influx.

### Infarct size

Several studies, though not all [3, 4], indicate that VNS limits infarct size, when started prior to [8, 21, 54], at [22, 25, 40] or halfway through [39, 48] the onset of myocardial ischemia (Table 4). The majority of these studies have been performed in rodents or rabbits, which are sympathetically dominant, and are therefore likely to have a different sympathico-vagal balance than larger mammalian species such as pigs and humans. Although it could be argued that this may enhance the effects of VNS in smaller animal species with low baseline vagal activity, recent studies observed that VNS produced similar reductions in infarct size in swine [39, 40], suggesting that its cardioprotective effects do not critically depend on basal vagal activity.

**Table 4:** Studies on the cardioprotective effects of vagal nerve stimulation

References	Species	I/R time	Start VNS	Duration VNS		Infarct size (% AR)	No-reflow (% IA)
*Pre/onset* *ischemia*							
Katare et al. [21]	Rat	30 min/2 h	5 min pre-ischemia	35 min	Sham	85 ± 3	–
					VNS	34 ± 2*	
Calvillo et al. [8]	Rat	30 min/24 h	5 min pre-ischemia	40 min	Sham	53 ± 5	–
					VNS	7 ± 1*	
Zhao et al. [54]	Rat	60 min/2 h	15 min pre-ischemia	75 min	Sham	47 ± 4	–
					VNS	27 ± 3*	
Kong et al. [25]	Rat	4 h/0 h	Onset ischemia	240 min	Sham	52 ± 2	–
					VNS	28 ± 2*	
Katare et al. [22]	Mouse	3 h/0 h	Onset ischemia	180 min	Sham	56 ± 1	–
					VNS	24 ± 2*	
Buchholz et al. [3]	Rabbit	30 min/3 h	10–15 min pre-ischemia	10 min	Sham	52 ± 4	–
					VNS	71 ± 4*	
Buchholz et al. [4]	Rabbit	45 min/4 h	10 min pre-ischemia	10 min	Sham	45 ± 2	–
					VNS	63 ± 3*	
					I-VNS	30 ± 3*	
Shinlapawittayatorn et al. [40]	Swine	60 min/2 h	Onset ischemia	180 min	Sham	46 ± 5	–
					VNS	19 ± 4*	
					I-VNS	5 ± 2*	
*Early/mid*-*ischemia*							
Shinlapawittayatorn et al. [39]	Swine	60 min/2 h	30 min into ischemia	150 min	Sham	46 ± 3	–
					I-VNS	19 ± 3*	
Wang et al. [48]	Rat	30 min/2 h	15 min into ischemia	30 min	Sham	72 ± 2	–
					VNS	47 ± 3*	
*Pre/onset* *reperfusion*							
Shinlapawittayatorn et al. [39]	Swine	60 min/2 h	Onset reperfusion	120 min	Sham	46 ± 3	–
					I-VNS	44 ± 3	
Uitterdijk et al.	Swine	45 min/2 h	5 min pre-reperfusion	20 min	Sham	67 ± 2	54 ± 6
					VNS	54 ± 5*	32 ± 6*

Data are mean ± SEM

*AR* area at risk, *IA* infarct area, *I/R* infarct/reperfusion time, *IS* infarct size, *VNS* vagal nerve stimulation, *I-VNS* intermittent vagal nerve stimulation (Buchholz: 10 s on/50 s off; Shinlapawittayatorn: 21 s on/30 s off)

* *P* < 0.05 versus corresponding sham. All studies were conducted in vivo except [13]

From the studies in Table 4, it is difficult to determine whether the protective effect of VNS occurred during ischemia or whether a reduction in lethal reperfusion injury contributed as well. Two studies in which no reperfusion was allowed [22, 25] suggest that at least part of the protective effect is targeted against ischemic cell death. Furthermore, a recent study reported that an intermittent VNS protocol failed to attenuate infarct size when started at the very onset of reperfusion [39], questioning whether VNS can protect against reperfusion injury. In view of the critical importance of the presence of an intervention during the golden first minute(s) of reperfusion [42], we hypothesized that continuous VNS started just prior to reperfusion might be effective against reperfusion injury. Indeed, we found that VNS, started a few minutes prior to reperfusion, was able to reduce myocardial infarct size, showing its cardioprotective potential against lethal reperfusion injury. The discordance between our findings and the study of Shinlapawittayatorn et al. [39] may well be due to the difference in VNS algorithm, i.e., intermittent VNS starting at the very onset of reperfusion [39] versus continuous VNS starting 5 min prior to reperfusion (Table 4), and may thus reflect the importance of full VNS during the golden first minute(s) of reperfusion [42].

The mechanism by which VNS limits infarct size is presently incompletely understood, but could involve indirect hemodynamic effects. Thus, the decrease in heart rate and the rate–pressure product could lower metabolic demand and modify reactive hyperemia during early reperfusion, thereby generating favorable (gentle) reperfusion conditions [32]. However, most evidence suggests that VNS-induced cardioprotection is independent of the reduction in heart rate. Thus, Calvillo et al. [8] showed that restoring the heart rate to baseline levels by atrial pacing did not affect cardioprotection by VNS, while several studies have shown a lack of correlation between the reduction in heart rate and the reduction in infarct size by VNS [8, 21, 40]. Those findings are supported by the lack of a significant correlation between the rate–pressure product and infarct size in the present study (Fig. 6). Moreover, coronary reactive hyperemia was not attenuated during VNS (Supplemental Figure S1), which may have been due to the opposing effects of the bradycardia-associated blunted metabolic stimulus for reactive hyperemia and the bradycardia-associated increase in diastolic perfusion time. Taken together, these findings suggest that the VNS-mediated cholinergic activation [8] and muscarinic receptor stimulation [40] protect against necrosis principally via a direct myocardial mechanism. Consequently, we studied the involvement of the reperfusion injury signaling kinase pathway, distal to the muscarinic receptor, by investigating the role of NO-synthase [9]. NO-synthase inhibition abolished VNS-mediated cardioprotection, at a time when the hemodynamic effects of VNS were unperturbed, indicating that cardiac NO-synthase activity was critical for VNS-mediated infarct-size and no-reflow reductions. These observations are in line with studies from our laboratory [28, 29] showing an important role for nitric oxide in cardioprotection against reperfusion injury, likely by limiting opening of the mitochondrial permeability transition pore [36, 39, 40]. Future studies are needed to further investigate the molecular underpinnings of VNS-mediated cardioprotection.

### No-reflow

Reperfusion following a prolonged period of myocardial ischemia is associated with microvascular obstruction, termed no-reflow [38]. No-reflow is the result of endothelial cell damage, deterioration of the glycocalyx, increased neutrophil plugging, micro-embolization, microvessel rupture and edema [15, 38]. No-reflow has been shown to be a strong clinical prognosticator for long-term outcome [33, 38], which is, at least in part, due to its close correlation with infarct size [18, 31, 45]. However, recent studies suggest that not only infarct size [30, 51], but also the extent of no-reflow [17, 33, 38] is an independent predictor of clinical outcome, which is supported by experimental studies reporting reductions in no-reflow by hypothermia [17] or pharmacological intervention [27], independent of a decrease in myocardial infarct size. These recent insights clearly suggest that novel strategies to limit no-reflow have significant therapeutic potential.

The present study is to our knowledge the first to investigate the effects of VNS on the extent of no-reflow. VNS markedly reduced no-reflow, which was accompanied by a reduction in recruitment of macrophages and neutrophils to the infarct area. These findings suggest that VNS modulates the regional immune response and are consistent with the activation of the cholinergic anti-inflammatory pathway by VNS [8, 20, 48]. VNS-induced reduction of no-reflow in the infarct area was prevented by NO-synthase inhibition in parallel with the abolition of infarct-size limitation, indicating that NO signaling is critical for the cardioprotective effects of VNS against both cardiomyocyte necrosis and microvascular obstruction.

### Methodological considerations

An intriguing observation in the present study was that the values for no-reflow of sham animals were considerably higher in the first (Fig. 2) than in the second (Table 3) series of experiments. The second series of experiments was performed approximately 1–2 years after completing the first series. Plotting all sham–control animals that underwent 45 min of ischemia and 120 min of reperfusion in the first series (2011 + 2012) and in the second series (2013 + 2014) demonstrates that the no-reflow area (and to a lesser extent infarct size) was significantly smaller in the latter period (Supplemental Figure S2). Although an explanation is not readily found (same supplier, same swine breed, same laboratory, same investigators), it should be noted that the first series was principally performed in the period of February–July, whereas the second series was performed in the period of August–January, suggesting that seasonal influences could be involved [24]. Although the present study does not allow identification of the exact mechanisms underlying these differences, the observations do emphasize the importance of time-matched sham–control experiments, as performed in the present study.

We also observed that while the occurrence of VF was high in both control + sham and LNNA-treated swine, there was a trend toward an increase in intractable VF from 27 % (3 out of 11) in sham animals to 53 % (9 out of 17) in LNNA-treated animals (*p* = 0.19). These findings are not readily explained, but there is evidence that loss of NO bioavailability increases the susceptibility to VF during ischemia–reperfusion [2, 6, 23]. Importantly, intractable VF invariably occurred prior to randomization to sham or VNS treatment in both control + sham and LNNA-treated animals and hence did not affect the study results.

A potential limitation of the present study is that we studied only the very early effects of VNS on infarct size and no-reflow in swine with acute myocardial infarction, with a follow-up limited to 2-h post-reperfusion. This time point was chosen in view of the demonstrated lack of development of infarct size and no-reflow over time between 2 and 5 h of reperfusion [16], so that we do not expect that infarct size and no-reflow would have evolved much further beyond the 2-h point. However, the 2-h reperfusion time was clearly insufficient to allow the VNS-mediated infarct-size reduction to translate into significant improvements in regional and global LV function. Future studies are required to investigate the long-term effects of infarct-size and no-reflow reductions by VNS on LV remodeling and function.

Another methodological consideration is the use of pentobarbital anesthesia. Pentobarbital is known to possess vagolytic properties, although this effect appears negligible in swine, as heart rates under pentobarbital anesthesia ([7]; present study) are not different from the heart rates we typically observe in awake resting swine [14]. Importantly, even if vagal tone is reduced, the ability to stimulate the vagal nerve and induce bradycardia remains principally intact [5], suggesting that pentobarbital’s vagolytic effects are centrally mediated and do not interfere with efferent vagal nerve stimulation.

In the present study, a custom-made coil to perform VNS was used, which may have contributed to a higher mA than what has been typically used (1–4 mA) in clinical studies [10, 35, 53] and in a previous study (1–4 mA; 30 Hz, pulse width 0.2 ms) from our laboratory in swine [7]. The latter was associated with reductions in heart rate of up to ~30 %. Those settings, which are similar to the settings used in VNS for the treatment of epilepsy and are well tolerated [37, 46], were similar in frequency and pulse duration as used in the present study (25 Hz, 0.3 ms), but the intensity used in the present study (10 mA) was significantly higher. The fact that we produced slightly smaller reductions in heart rate at higher VNS intensities may reflect the custom coil versus the clinical grade coil that we previously had access to. It is important to note that this was a proof-of-concept study that did not test a clinically approved device, but investigated the efficacy of VNS started just prior to reperfusion and continued for only 15 min into reperfusion, in which we were able to demonstrate cardioprotection, despite a modest 20 % reduction in heart rate.

Another limitation of the present study is that only a single VNS protocol was studied and hence it is likely that other VNS algorithms may afford greater cardioprotection [25]. Optimization of the VNS protocol may include changes in stimulation frequency [1] and extending the duration of stimulation beyond 15 min of reperfusion.

### Clinical implications

The present study demonstrates that VNS starting just prior to and lasting only 15 min into reperfusion is effective in reducing infarct size and no-reflow in a large animal model of reperfused STEMI. VNS thus appears to be an attractive potential adjuvant therapy to limit reperfusion injury in patients with STEMI. The current technology includes implantable stimulators [47]; but with the ongoing development of transvenous and transdermal [49, 50] approaches that do not require dissection of the vagal nerve, investigation of VNS in the clinical setting of STEMI appears warranted.

## Conclusions

The present study in a porcine model of acute myocardial infarction demonstrated that vagal nerve stimulation (VNS) started just prior to reperfusion and continued during early reperfusion-limited infarct size and the extent of no-reflow. The cardioprotection appeared to occur independently of the VNS-induced decrease in heart rate and was not associated with a reduction in systemic markers of inflammation. However, VNS did result in reduced neutrophil and macrophage influx into the infarct area. Finally, NO-synthase activity was required for the VNS-induced limitation in infarct size and no-reflow. Taken together, our findings indicate that VNS is a promising novel adjunctive therapy to limit reperfusion injury.

## Electronic supplementary material

Supplementary material 1 (TIFF 319 kb)

Supplementary material 2 (TIFF 1622 kb)

Supplementary material 3 (DOCX 30 kb)

Supplementary material 4 (DOCX 20 kb)

## References

[1] Bonaz B, Picq C, Sinniger V, Mayol JF, Clarencon D (2013). Vagus nerve stimulation: from epilepsy to the cholinergic anti-inflammatory pathway. Neurogastroenterol Motil.

[2] Brutsaert DL (2003). Cardiac endothelial-myocardial signalling: its role in cardiac growth, contractile performance, and rhythmicity. Physiol Rev.

[3] Buchholz B, Donato M, Perez V, Deutsch AC, Hocht C, Del Mauro JS, Rodriguez M, Gelpi RJ (2014). Changes in the loading conditions induced by vagal stimulation modify the myocardial infarct size through sympathetic-parasympathetic interactions. Pflügers Arch.

[4] Buchholz B, Donato M, Perez V, Ivalde FC, Hocht C, Buitrago E, Rodriguez M, Gelpi RJ (2012). Preischemic efferent vagal stimulation increases the size of myocardial infarction in rabbits. Role of the sympathetic nervous system. Int J Cardiol.

[5] Buckley NM, Gootman PM, Brazeau P, Matanic BP, Frasier ID, Gentles EL (1979). Cardiovascular function in anesthetized miniature swine. Lab Anim Sci.

[6] Burger DE, Lu X, Lei M, Xiang FL, Hammoud L, Jiang M, Wang H, Jones DL, Sims SM, Feng Q (2009). Neuronal nitric oxide synthase protects against myocardial infarction-induced ventricular arrhythmia and mortality in mice. Circulation.

[7] Buschman HP, Storm CJ, Duncker DJ, Verdouw PD, van der Aa HE, van der Kemp P (2006). Heart rate control via vagus nerve stimulation. Neuromodulation.

[8] Calvillo L, Vanoli E, Andreoli E, Besana A, Omodeo E, Gnecchi M, Zerbi P, Vago G, Busca G, Schwartz PJ (2011). Vagal stimulation, through its nicotinic action, limits infarct size and the inflammatory response to myocardial ischemia and reperfusion. J Cardiovasc Pharmacol.

[9] Davidson SM, Hausenloy D, Duchen MR, Yellon DM (2006). Signalling via the reperfusion injury signalling kinase (RISK) pathway links closure of the mitochondrial permeability transition pore to cardioprotection. Int J Biochem Cell Biol.

[10] De Ferrari GM, Crijns HJ, Borggrefe M, Milasinovic G, Smid J, Zabel M, Gavazzi A, Sanzo A, Dennert R, Kuschyk J, Raspopovic S, Klein H, Swedberg K, Schwartz PJ (2011). Chronic vagus nerve stimulation: a new and promising therapeutic approach for chronic heart failure. Eur Heart J.

[11] de Zeeuw S, Trines SA, Krams R, Verdouw PD, Duncker DJ (2000). Cardiovascular profile of the calcium sensitizer EMD 57033 in open-chest anaesthetized pigs with regionally stunned myocardium. Br J Pharmacol.

[12] Dirksen MT, Laarman GJ, Simoons ML, Duncker DJGM (2007). Reperfusion injury in humans: a review of clinical trials on reperfusion injury inhibitory strategies. Cardiovasc Res.

[13] Duncker DJ, Klassen CL, Ishibashi Y, Herrlinger SH, Pavek TJ, Bache RJ (1996). Effect of temperature on myocardial infarction in swine. Am J Physiol.

[14] Duncker DJ, Stubenitsky R, Tonino PA, Verdouw PD (2000). Nitric oxide contributes to the regulation of vasomotor tone but does not modulate O_2_-consumption in exercising swine. Cardiovasc Res.

[15] Galasso G, Schiekofer S, D’Anna C, Di Gioia G, Piccolo R, Niglio T, De Rosa R, Strisciuglio T, Cirillo P, Piscione F, Trimarco B (2014). No-reflow phenomenon: pathophysiology, diagnosis, prevention, and treatment. A review of the current literature and future perspectives. Angiology.

[16] Hale SL, Dae MW, Kloner RA (2003). Hypothermia during reperfusion limits ‘no-reflow’ injury in a rabbit model of acute myocardial infarction. Cardiovasc Res.

[17] Hale SL, Herring MJ, Kloner RA (2013). Delayed treatment with hypothermia protects against the no-reflow phenomenon despite failure to reduce infarct size. J Am Heart Assoc.

[18] Heusch G, Kleinbongard P, Skyschally A (2013). Myocardial infarction and coronary microvascular obstruction: an intimate, but complicated relationship. Basic Res Cardiol.

[19] Ibanez B, Heusch G, Ovize M, Van de Werf F (2015). Evolving therapies for myocardial ischemia/reperfusion injury. J Am Coll Cardiol.

[20] Johnston GR, Webster NR (2009). Cytokines and the immunomodulatory function of the vagus nerve. Br J Anaesth.

[21] Katare RG, Ando M, Kakinuma Y, Arikawa M, Handa T, Yamasaki F, Sato T (2009). Vagal nerve stimulation prevents reperfusion injury through inhibition of opening of mitochondrial permeability transition pore independent of the bradycardiac effect. J Thorac Cardiovasc Surg.

[22] Katare RG, Ando M, Kakinuma Y, Arikawa M, Yamasaki F, Sato T (2010). Differential regulation of TNF receptors by vagal nerve stimulation protects heart against acute ischemic injury. J Mol Cell Cardiol.

[23] Kawahara K, Takase M, Yamauchi Y (2003). Increased vulnerability to ischemia/reperfusion-induced ventricular tachyarrhythmias by pre-ischemic inhibition of nitric oxide synthase in isolated rat hearts. Cardiovasc Pathol.

[24] Kloner RA, Das S, Poole WK, Perrit R, Muller J, Cannon CP, Braunwald E (2001). Seasonal variation of myocardial infarct size. Am J Cardiol.

[25] Kong SS, Liu JJ, Hwang TC, Yu XJ, Zhao M, Zhao M, Yuan BX, Lu Y, Kang YM, Wang B, Zang WJ (2012). Optimizing the parameters of vagus nerve stimulation by uniform design in rats with acute myocardial infarction. Plos One.

[26] Koning MM, Gho BC, van Klaarwater E, Opstal RL, Duncker DJ, Verdouw PD (1996). Rapid ventricular pacing produces myocardial protection by nonischemic activation of K^+^_ATP_ channels. Circulation.

[27] Li XD, Yang YJ, Geng YJ, Jin C, Hu FH, Zhao JL, Zhang HT, Cheng YT, Qian HY, Wang LL, Zhang BJ, Wu YL (2010). Tongxinluo reduces myocardial no-reflow and ischemia-reperfusion injury by stimulating the phosphorylation of eNOS via the PKA pathway. Am J Physiol Heart Circ Physiol.

[28] Manintveld OC, te Lintel Hekkert M, van der Ploeg NT, Verdouw PD, Duncker DJ (2009). Interaction between pre- and postconditioning in the in vivo rat heart. Exp Biol Med (Maywood).

[29] Manintveld OC, Sluiter W, Dekkers DH, te Lintel Hekkert M, Lamers JM, Verdouw PD, Duncker DJ (2011). Involvement of reperfusion injury salvage kinases in preconditioning depends critically on the preconditioning stimulus. Exp Biol Med (Maywood).

[30] Masci PG, Ganame J, Francone M, Desmet W, Lorenzoni V, Iacucci I, Barison A, Carbone I, Lombardi M, Agati L, Janssens S, Bogaert J (2011). Relationship between location and size of myocardial infarction and their reciprocal influences on post-infarction left ventricular remodelling. Eur Heart J.

[31] Mewton N, Thibault H, Roubille F, Lairez O, Rioufol G, Sportouch C, Sanchez I, Bergerot C, Cung TT, Finet G, Angoulvant D, Revel D, Bonnefoy-Cudraz E, Elbaz M, Piot C, Sahraoui I, Croisille P, Ovize M (2013). Postconditioning attenuates no-reflow in STEMI patients. Basic Res Cardiol.

[32] Musiolik J, van Caster P, Skyschally A, Boengler K, Gres P, Schulz R, Heusch G (2010). Reduction of infarct size by gentle reperfusion without activation of reperfusion injury salvage kinases in pigs. Cardiovasc Res.

[33] Ndrepepa G, Tiroch K, Fusaro M, Keta D, Seyfarth M, Byrne RA, Pache J, Alger P, Mehilli J, Schomig A, Kastrati A (2010). 5-Year prognostic value of no-reflow phenomenon after percutaneous coronary intervention in patients with acute myocardial infarction. J Am Coll Cardiol.

[34] Niccoli G, Burzotta F, Galiuto L, Crea F (2009). Myocardial no-reflow in humans. J Am Coll Cardiol.

[35] Premchand RK, Sharma K, Mittal S, Monteiro R, Dixit S, Libbus I, DiCarlo LA, Ardell JL, Rector TS, Amurthur B, KenKnight BH, Anand IS (2014). Autonomic regulation therapy via left or right cervical vagus nerve stimulation in patients with chronic heart failure: results of the ANTHEM-HF trial. J Card Fail.

[36] Reffelmann T, Kloner RA (2002). Microvascular reperfusion injury: rapid expansion of anatomic no reflow during reperfusion in the rabbit. Am J Physiol Heart Circ Physiol.

[37] Rutecki P (1990). Anatomical, physiological, and theoretical basis for the antiepileptic effect of vagus nerve stimulation. Epilepsia.

[38] Schwartz BG, Kloner RA (2012). Coronary no reflow. J Mol Cell Cardiol.

[39] Shinlapawittayatorn K, Chinda K, Palee S, Surinkaew S, Kumfu S, Kumphune S, Chattipakorn S, KenKnight BH, Chattipakorn N (2014). Vagus nerve stimulation initiated late during ischemia, but not reperfusion, exerts cardioprotection via amelioration of cardiac mitochondrial dysfunction. Heart Rhythm.

[40] Shinlapawittayatorn K, Chinda K, Palee S, Surinkaew S, Thunsiri K, Weerateerangkul P, Chattipakorn S, KenKnight BH, Chattipakorn N (2013). Low-amplitude, left vagus nerve stimulation significantly attenuates ventricular dysfunction and infarct size through prevention of mitochondrial dysfunction during acute ischemia-reperfusion injury. Heart Rhythm.

[41] Silber S, Albertsson P, Aviles FF, Camici PG, Colombo A, Hamm C, Jorgensen E, Marco J, Nordrehaug JE, Ruzyllo W, Urban P, Stone GW, Wijns W (2005). Guidelines for percutaneous coronary interventions—the task force for percutaneous coronary interventions of the European Society of Cardiology. Eur Heart J.

[42] Skyschally A, van Caster P, Iliodromitis EK, Schulz R, Kremastinos DT, Heusch G (2009). Ischemic postconditioning: experimental models and protocol algorithms. Basic Res Cardiol.

[43] Steg PG, James SK, Atar D, Badano LP, Blomstrom-Lundqvist C, Borger MA, Di Mario C, Dickstein K, Ducrocq G, Fernandez-Aviles F, Gershlick AH, Giannuzzi P, Halvorsen S, Huber K, Juni P, Kastrati A, Knuuti J, Lenzen MJ, Mahaffey KW, Valgimigli M, van ‘t Hof A, Widimsky P, Zahger D, Task Force on the management of ST-segment elevation acute myocardial infarction of the European Society of Cardiology (ESC) (2012). ESC Guidelines for the management of acute myocardial infarction in patients presenting with ST-segment elevation. Eur Heart J.

[44] Te Lintel Hekkert M, Dube GP, Regar E, de Boer M, Vranckx P, van der Giessen WJ, Serruys PW, Duncker DJ (2010). Preoxygenated hemoglobin-based oxygen carrier HBOC-201 annihilates myocardial ischemia during brief coronary artery occlusion in pigs. Am J Physiol Heart Circ Physiol.

[45] Uitterdijk A, Sneep S, van Duin R, Krabbendam-Peters I, Gorsse-Bakker C, Duncker DJ, van der Giessen WJ, van Beusekom HM (2013). Serial measurement of hFABP and high sensitivity Troponin I post PCI in STEMI. How fast and accurate can myocardial infarct size and no-reflow be predicted?. Am J Physiol Heart Circ Physiol.

[46] Uthman BM, Wilder BJ, Penry JK, Dean C, Ramsay RE, Reid SA, Hammond EJ, Tarver WB, Wernicke JF (1993). Treatment of epilepsy by stimulation of the vagus nerve. Neurology.

[47] Wagner D, Shelton R, Adams D, Garlie J, Rhee JK, Chen PS, Lin SF (2012). An interactive implantable vagal nerve stimulator for real-time modulation of cardiac autonomic control. Conf Proc IEEE Eng Med Biol Soc.

[48] Wang Q, Cheng Y, Xue FS, Yuan YJ, Xiong J, Li RP, Liao X, Liu JH (2012). Postconditioning with vagal stimulation attenuates local and systemic inflammatory responses to myocardial ischemia reperfusion injury in rats. Inflamm Res.

[49] Wang Z, Yu L, Huang B, Wang S, Liao K, Saren G, Zhou X, Jiang H (2015). Low-level transcutaneous electrical stimulation of the auricular branch of vagus nerve ameliorates left ventricular remodeling and dysfunction by downregulation of matrix metalloproteinase 9 and transforming growth factor beta1. J Cardiovasc Pharmacol.

[50] Wang Z, Yu L, Wang S, Huang B, Liao K, Saren G, Tan T, Jiang H (2014). Chronic intermittent low-level transcutaneous electrical stimulation of auricular branch of vagus nerve improves left ventricular remodeling in conscious dogs with healed myocardial infarction. Circ Heart Fail.

[51] Wu E, Ortiz JT, Tejedor P, Lee DC, Bucciarelli-Ducci C, Kansal P, Carr JC, Holly TA, Lloyd-Jones D, Klocke FJ, Bonow RO (2008). Infarct size by contrast enhanced cardiac magnetic resonance is a stronger predictor of outcomes than left ventricular ejection fraction or end-systolic volume index: prospective cohort study. Heart.

[52] Yellon DM, Hausenloy DJ (2007). Myocardial reperfusion injury. N Engl J Med.

[53] Zannad F, De Ferrari GM, Tuinenburg AE, Wright D, Brugada J, Butter C, Klein H, Stolen C, Meyer S, Stein KM, Ramuzat A, Schubert B, Daum D, Neuzil P, Botman C, Castel MA, D’Onofrio A, Solomon SD, Wold N, Ruble SB (2015). Chronic vagal stimulation for the treatment of low ejection fraction heart failure: results of the NEural Cardiac TherApy foR Heart Failure (NECTAR-HF) randomized controlled trial. Eur Heart J.

[54] Zhao M, He X, Bi XY, Yu XJ, Wier WG, Zang WJ (2013). Vagal stimulation triggers peripheral vascular protection through the cholinergic anti-inflammatory pathway in a rat model of myocardial ischemia/reperfusion. Basic Res Cardiol.

