# Supplementary figures and images for: Vagal nerve stimulation started just prior to reperfusion limits infarct size and no-reflow
Source: Basic Res Cardiol. 2015 Aug 26;110(5):51. doi: 10.1007/s00395-015-0508-3 (PMC4549380; doi:10.1007/s00395-015-0508-3)

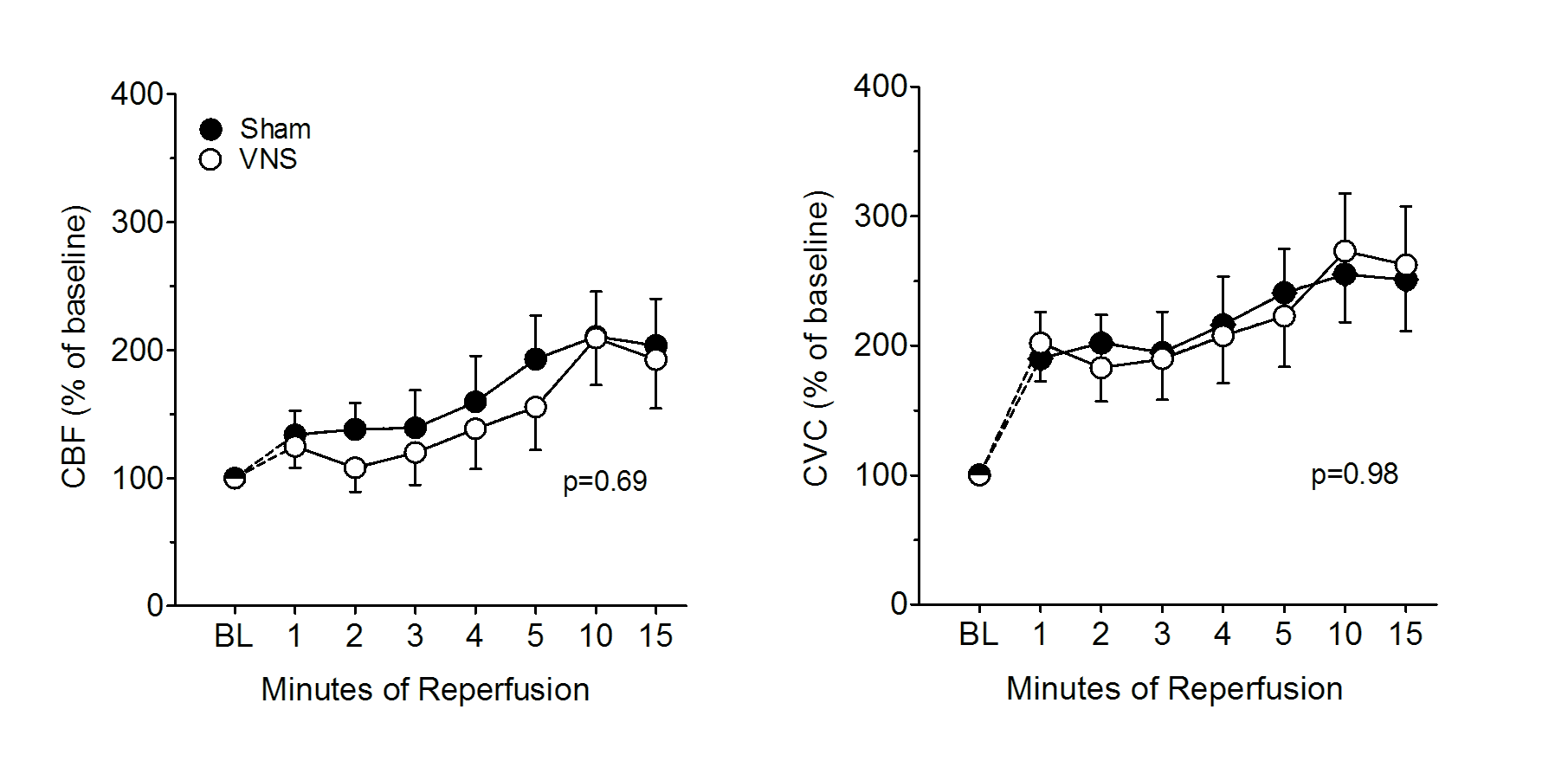

Supplement: Supplementary file 1 — Supplementary material 1 (TIFF 319 kb) [file 395_2015_508_MOESM1_ESM.tif]

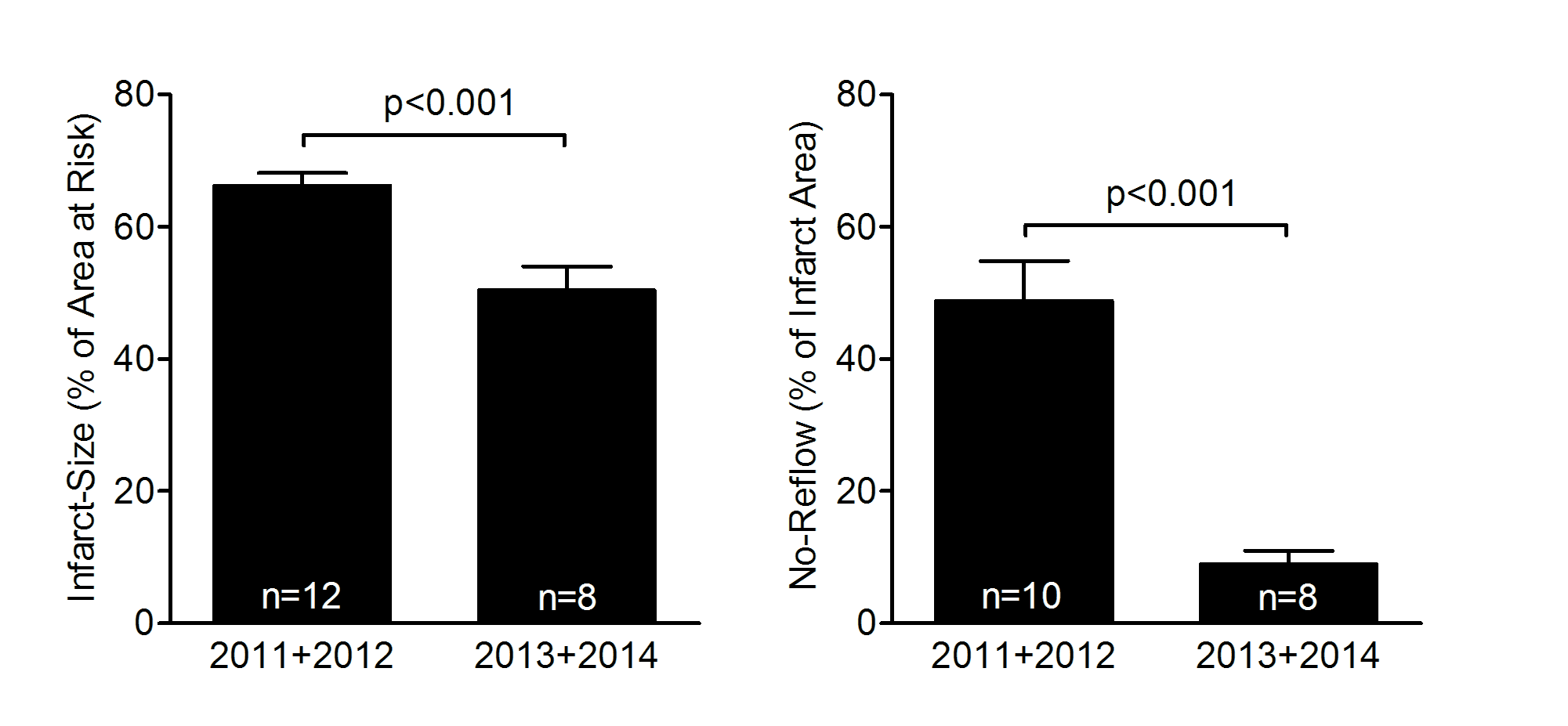

Supplement: Supplementary file 2 — Supplementary material 2 (TIFF 1622 kb) [file 395_2015_508_MOESM2_ESM.tif]
