# Supplementary material for: Vagal nerve stimulation started just prior to reperfusion limits infarct size and no-reflow
Source: Basic Res Cardiol. 2015 Aug 26;110(5):51. doi: 10.1007/s00395-015-0508-3 (PMC4549380; doi:10.1007/s00395-015-0508-3)
Supplement: Supplementary file 3 — Supplementary material 3 (DOCX 30 kb) [file 395_2015_508_MOESM3_ESM.docx]

| **Table S1. Systemic Hemodynamics and Global and Regional Left Ventricular Function** | | | | | | | | | | | | | | | | | | | | |
| --- | --- | --- | --- | --- | --- | --- | --- | --- | --- | --- | --- | --- | --- | --- | --- | --- | --- | --- | --- | --- |
|  | | | | | **Coronary Artery Occlusion** | | | | | | | |  |  | **Reperfusion** | | | | | |
|  | | **Baseline** | | | **40 min** | | | **45 min** | | |  |  | | **15 min** | | | | **120 min** | | |
| *Systemic Hemodynamics* | | | | | | | | | | | | | | | | | | | | |
| HR (bpm) | Sham | 100 | ± | 5 | 103 | ± | 3 | 103 | ± | 3 | | |  | 100 | | ± | 4 | 112 | ± | 6* |
|  | LNNA+Sham | 100 | ± | 14 | 96 | ± | 10 | 95 | ± | 10 | | |  | 92 | | ± | 9 | 97 | ± | 8 |
|  | LNNA+VNS | 96 | ± | 4 | 95 | ± | 3 | 78 | ± | 3*†§ | | |  | 86 | | ± | 4†§ | 114 | ± | 3*†§ |
| MAP (mmHg) | Sham | 85 | ± | 3 | 67 | ± | 4* | 64 | ± | 3* | | |  | 64 | | ± | 3* | 60 | ± | 4* |
|  | LNNA+Sham | 104 | ± | 9 | 68 | ± | 7* | 66 | ± | 7* | | |  | 56 | | ± | 7*‡ | 77 | ± | 13* |
|  | LNNA+VNS | 111 | ± | 13 | 95 | ± | 17* | 76 | ± | 15*†§ | | |  | 66 | | ± | 17*† | 91 | ± | 10* |
| CO (L/min) | Sham | 3.3 | ± | 0.3 | 2.8 | ± | 0.2* | 2.8 | ± | 0.2* | | |  | 2.9 | | ± | 0.2* | 2.1 | ± | 0.3*† |
|  | LNNA+Sham | 3.2 | ± | 0.2 | 2.6 | ± | 0.2* | 2.6 | ± | 0.2* | | |  | 2.3 | | ± | 0.3* | 2.3 | ± | 0.2* |
|  | LNNA+VNS | 3.2 | ± | 0.2 | 2.6 | ± | 0.1* | 2.1 | ± | 0.1*†§ | | |  | 1.8 | | ± | 0.3*†§ | 1.7 | ± | 0.3*† |
| SV (mL/beat) | Sham | 33 | ± | 2 | 28 | ± | 2* | 27 | ± | 2* | | |  | 29 | | ± | 2 | 19 | ± | 3*† |
|  | LNNA+Sham | 34 | ± | 4 | 28 | ± | 3 | 28 | ± | 3* | | |  | 26 | | ± | 4* | 24 | ± | 2* |
|  | LNNA+VNS | 33 | ± | 2 | 28 | ± | 1* | 27 | ± | 2* | | |  | 20 | | ± | 3*† | 15 | ± | 3*† |
| *Global and Regional LV Function* | | | | | | | | | | | | | | | | | | | | |
| LVSP (mmHg) | Sham | 99 | ± | 3 | 80 | ± | 4* | 77 | ± | 3* | | |  | 78 | | ± | 3* | 70 | ± | 4* |
|  | LNNA+Sham | 118 | ± | 7 | 80 | ± | 7* | 79 | ± | 6* | | |  | 69 | | ± | 7*‡ | 90 | ± | 13* |
|  | LNNA+VNS | 124 | ± | 15 | 106 | ± | 17* | 86 | ± | 14*†§ | | |  | 74 | | ± | 15*† | 98 | ± | 9* |
| LVdP/dt_P=40_ (mmHg/s) | Sham | 1350 | ± | 130 | 990 | ± | 90* | 950 | ± | 90* | | |  | 980 | | ± | 80* | 770 | ± | 100* |
|  | LNNA+Sham | 1500 | ± | 310 | 940 | ± | 90* | 930 | ± | 100* | | |  | 790 | | ± | 140* | 1160 | ± | 310* |
|  | LNNA+VNS | 1330 | ± | 110 | 1060 | ± | 130 | 860 | ± | 170* | | |  | 1050 | | ± | 250 | 1030 | ± | 120 |
| LVEDP (mmHg) | Sham | 12 | ± | 1 | 16 | ± | 1* | 15 | ± | 1* | | |  | 16 | | ± | 1* | 12 | ± | 1† |
|  | LNNA+Sham | 12 | ± | 1 | 13 | ± | 1 | 13 | ± | 1 | | |  | 13 | | ± | 1‡ | 13 | ± | 2 |
|  | LNNA+VNS | 11 | ± | 1 | 13 | ± | 1 | 12 | ± | 1 | | |  | 12 | | ± | 1 | 11 | ± | 1 |
| SS_LAD_ (%) | Sham | 18.8 | ± | 0.6 | -6.8 | ± | 3.0* | -4.6 | ± | 1.9* | | |  | 4.2 | | ± | 2.4*† | 1.1 | ± | 1.8*† |
|  | LNNA+Sham | 18.9 | ± | 1.7 | -7.2 | ± | 1.3* | -6.8 | ± | 1.8* | | |  | 1.9 | | ± | 3.5*† | -1.4 | ± | 3.3* |
|  | LNNA+VNS | 17.1 | ± | 2.3 | -2.9 | ± | 2.6* | -0.6 | ± | 2.4* | | |  | 2.7 | | ± | 1.2* | -0.5 | ± | 1.8* |
| SS_LCx_ (%) | Sham | 19.9 | ± | 1.0 | 20.0 | ± | 2.3 | 18.5 | ± | 1.2 | | |  | 18.5 | | ± | 1.7 | 13.3 | ± | 1.3*† |
|  | LNNA+Sham | 15.9 | ± | 2.6 | 12.5 | ± | 2.8 | 12.2 | ± | 2.7 | | |  | 11.0 | | ± | 1.3 | 7.9 | ± | 1.8* |
|  | LNNA+VNS | 15.1 | ± | 1.6 | 14.9 | ± | 2.0 | 14.9 | ± | 1.8 | | |  | 13.8 | | ± | 1.8 | 8.9 | ± | 2.7*† |
| PSS_LAD_(%) | Sham | 1.3 | ± | 0.4 | 14.0 | ± | 2.2* | 11.4 | ± | 1.2* | | |  | 4.2 | | ± | 1.6† | 5.0 | ± | 1.7† |
|  | LNNA+Sham | 2.9 | ± | 0.7 | 12.7 | ± | 1.3* | 11.7 | ± | 1.4* | | |  | 4.1 | | ± | 3.4† | 7.5 | ± | 3.9 |
|  | LNNA+VNS | 3.7 | ± | 1.3 | 9.9 | ± | 1.7* | 6.7 | ± | 1.7 | | |  | 3.1 | | ± | 1.3† | 5.4 | ± | 1.5 |
| PSS_LCx_ (%) | Sham | 0.1 | ± | 0.1 | 0.5 | ± | 0.2 | 0.6 | ± | 0.3 | | |  | 0.3 | | ± | 0.2 | 0.3 | ± | 0.1 |
|  | LNNA+Sham | 1.1 | ± | 0.4 | 2.4 | ± | 0.7 | 2.3 | ± | 0.7 | | |  | 1.2 | | ± | 0.5 | 1.8 | ± | 0.6 |
|  | LNNA+VNS | 2.6 | ± | 1.4 | 2.4 | ± | 1.0 | 1.6 | ± | 0.9 | | |  | 1.3 | | ± | 1.1 | 2.9 | ± | 1.2 |
| Data are mean ± SEM; Sham group, n=8; LNNA+Sham group, n=5; LNNA+VNS group, n=6. Except for PSS_LCx_ LNNA+VNS vs Sham (p=0.024), no differences between groups at baseline; *p<0.05 vs. corresponding baseline; †p<0.05 vs. corresponding 40 min CAO; ‡p<0.05 change by LNNA+Sham vs. Sham; §p<0.05 change by LNNA+VNS vs. LNNA+Sham. CO = cardiac output; dP/dt_P=40_ = rate of rise in left ventricular (LV) pressure during LV pressure of 40 mmHg; HR = heart rate; LVEDP = left ventricular end-diastolic pressure; LVSP = left ventricular systolic pressure; MAP = mean arterial pressure; PSS = post-systolic shortening; SS = systolic shortening; SV = stroke volume; VNS = Vagal nerve stimulation. | | | | | | | | | | | | | | | | | | | | |
