# Supplementary material for: Vagal nerve stimulation started just prior to reperfusion limits infarct size and no-reflow
Source: Basic Res Cardiol. 2015 Aug 26;110(5):51. doi: 10.1007/s00395-015-0508-3 (PMC4549380; doi:10.1007/s00395-015-0508-3)
Supplement: Supplementary file 4 — Supplementary material 4 (DOCX 20 kb) [file 395_2015_508_MOESM4_ESM.docx]

| **Table S2. Regional Coronary Blood Flow and Myocardial Metabolism** | | | | | | | | | | | | |
| --- | --- | --- | --- | --- | --- | --- | --- | --- | --- | --- | --- | --- |
|  | |  | | | | **Reperfusion** | | | | | | |
|  | |  | **Baseline** | | | **15 min** | | | | **120 min** | | |
| CBF (mL/min) | Sham | 16 | | ± | 2 | 33 | ± | 4 | 22 | | ± | 4 |
|  | LNNA+Sham | 13 | | ± | 1 | 17 | ± | 3 | 29 | | ± | 9 |
|  | LNNA+VNS | 22 | | ± | 1 | 28 | ± | 13 | 27 | | ± | 3 |
| CBF (mL/beat) | Sham | 0.17 | | ± | 0.02 | 0.34 | ± | 0.04 | 0.20 | | ± | 0.03 |
|  | LNNA+Sham | 0.15 | | ± | 0.03 | 0.20 | ± | 0.05 | 0.29 | | ± | 0.06 |
|  | LNNA+VNS | 0.23 | | ± | 0.02 | 0.30 | ± | 0.12 | 0.24 | | ± | 0.03 |
| MVO_2_ (µmol/min) | Sham | 66 | | ± | 4 | 41 | ± | 3* | 17 | | ± | 3* |
|  | LNNA+Sham | 61 | | ± | 5 | 23 | ± | 4* | 46 | | ± | 10† |
|  | LNNA+VNS | 98 | | ± | 8 | 38 | ± | 4* | 35 | | ± | 5*‡ |
| MVO_2_ per beat (µmol/beat) | Sham | 0.67 | | ± | 0.06 | 0.42 | ± | 0.04* | 0.16 | | ± | 0.03* |
|  | LNNA+Sham | 0.66 | | ± | 0.11 | 0.26 | ± | 0.05* | 0.48 | | ± | 0.11 |
|  | LNNA+VNS | 1.03 | | ± | 0.09 | 0.42 | ± | 0.05* | 0.31 | | ± | 0.04*‡ |
| CVC (ml/min/mmHg) | Sham | 0.20 | | ± | 0.02 | 0.51 | ± | 0.05* | 0.38 | | ± | 0.08* |
|  | LNNA+Sham | 0.13 | | ± | 0.02 | 0.32 | ± | 0.06 | 0.36 | | ± | 0.05 |
|  | LNNA+VNS | 0.21 | | ± | 0.03 | 0.38 | ± | 0.09 | 0.30 | | ± | 0.03 |
| Lactate Production (µmol/L/min) | Sham | -11 | | ± | 1 | 7 | ± | 4* | -7 | | ± | 2 |
|  | LNNA+Sham | -11 | | ± | 2 | 7 | ± | 4* | -6 | | ± | 7 |
|  | LNNA+VNS | -21 | | ± | 3 | -3 | ± | 4* | -18 | | ± | 5 |
| O_2_ Extraction (%) | Sham | 70 | | ± | 2 | 19 | ± | 2* | 25 | | ± | 4* |
|  | LNNA+Sham | 79 | | ± | 1 | 24 | ± | 3* | 33 | | ± | 15* |
|  | LNNA+VNS | 80 | | ± | 1 | 40 | ± | 9* | 24 | | ± | 8* |
| Lactate Extraction (%) | Sham | 24 | | ± | 3 | -5 | ± | 3* | 12 | | ± | 2* |
|  | LNNA+Sham | 28 | | ± | 3 | -13 | ± | 6* | -1 | | ± | 5* |
|  | LNNA+VNS | 38 | | ± | 7 | 2 | ± | 4* | 13 | | ± | 2* |
| Data are mean ± SEM; Sham group, n=8; LNNA+Sham, n=5; LNNA+VNS group, n=6. Except for MVO_2_ (LNNA+VNS vs Sham, p<0.001 and LNNA+VNS vs LNNA+Sham, p<0.001) and MVO_2_ per beat ( LNNA+VNS vs Sham, p<0.001 and LNNA+VNS vs LNNA+Sham, p=0.001), no differences between groups at baseline; *p<0.05 vs. corresponding baseline; †p<0.05 change by LNNA+Sham vs Sham; ‡p<0.05 change by LNNA+VNS vs LNNA+Sham. CBF = coronary blood flow; CVC = coronary vascular conductance (CBF/MAP); MVO_2_ = myocardial oxygen consumption; O_2_ Extraction = myocardial oxygen extraction. | | | | | | | | | | | | |
